# Primary, Secondary Metabolites, H_2_O_2_, Malondialdehyde and Photosynthetic Responses of *Orthosiphon stimaneus* Benth. to Different Irradiance Levels

**DOI:** 10.3390/molecules17021159

**Published:** 2012-01-27

**Authors:** Mohd Hafiz Ibrahim, Hawa Z. E. Jaafar

**Affiliations:** Department of Crop Science, Faculty of Agriculture, University Putra Malaysia, 43400 Serdang, Selangor, Malaysia; Email: mhafizphd@yahoo.com (M.H.I.)

**Keywords:** light intensity, total non-structural carbohydrate, carbon-based secondary metabolites, lipid peroxidation, total soluble sugar, protein competition model, C/N ratio

## Abstract

The resource availability hypothesis predicts an increase in the allocation to secondary metabolites when carbon gain is improved relative to nutrient availability, which normally occurs during periods of low irradiance. The present work was carried out to confirm this hypothesis by investigating the effects of decreasing irradiance on the production of plant secondary metabolites (flavonoids and phenolics) in the herbal plant *Orthosiphon stamineus*, and to characterize this production by carbohydrate, H_2_O_2_, and malondialdehyde (MDA) levels, net photosynthesis, leaf chlorophyll content and carbon to nitrogen ratio (C/N). Four levels of irradiance (225, 500, 625 and 900 µmol/m^2^/s) were imposed onto two-week old seedlings for 12 weeks in a randomized complete block design experiment. Peak production of total flavonoids, phenolics, soluble sugar, starch and total non-structural carbohydrate ocurred under low irradiance of 225 µmol/m^2^/s, and decreased with increasing irradiance. The up-regulation of secondary metabolites could be explained by the concomitant increases in H_2_O_2_ and MDA activities under low irradiance. This condition also resulted in enhanced C/N ratio signifying a reduction in nitrogen levels, which had established significant negative correlations with net photosynthesis, total biomass and total chlorophyll content, indicating the possible existence of a trade-off between growth and secondary metabolism under low irradiance with reduced nitrogen content. The competition between total chlorophyll and secondary metabolites production, as exhibited by the negative correlation coefficient under low irradiance, also suggests a sign of gradual switch of investment from chlorophyll to polyphenols production.

## 1. Introduction

*Orthosiphon stamineus* Benth. (Lamiaceae), commonly known as misai kucing, is a popular medicinal plant in Southeast Asia and is widely used for the treatment of eruptive fever, epilepsy, gallstones, hepatitis, rheumatism, hypertension, syphilis and renal calculus [1]. In Malaysia and Indonesia, *Orthosiphon stamineus* leaves are prepared in the form of infusion, popularly known as Java tea, and consumed as a beverage to improve overall health and for treatment of kidney, bladder inflammation, gout and diabetes [1]. Several classes of compounds have been identified from this plant, including flavonoids, terpenoids, saponins, hexoses, organic acids, caffeic acids derivatives, chromene and myo-inositol [2,3,4,5,6]. Among these compounds, the polyphenol derivatives were found to possess potential therapeutic properties as they were shown to exert diuretic and uricosuric actions in rats [2]. The therapeutic effects of *Orthosiphon stimaneus* have been ascribed mainly to its polyphenols, the most dominant constituent in the leaf, which have been reported to be effective in reducing oxidative stress by inhibiting the formation of lipid peroxidation products in biological systems [7]. Polyphenols are very important in the control and prevention of tissue damage done by activated oxygen species due to their antioxidative effects [8].

The concentration of polyphenols was found to be influenced by environmental conditions such as light intensity, carbon dioxide levels, temperature, fertilization, and biotic and abiotic factors, which can alter the concentration of these active constituents [9]. Irradiance is known to regulate not only plant growth and development, but also the biosynthesis of both primary and secondary metabolites [10,11]. Phenolics biosynthesis requires irradiance or is enhanced by irradiance, and flavonoids formation is absolutely irradiance dependent where its biosynthetic rate is related to irradiance intensity and density [12]. Different plants have different responses to irradiance intensity that result in differences in their production of secondary metabolites. In general, the resource availability hypothesis [13] predicts a decline in allocation to secondary metabolites when carbon gain is limited relative to nutrient availability, which normally occurs during periods of low irradiance. This prediction was based on the effects of environmental conditions on carbon-nutrient balance [14]. Secondary metabolites production might increase [15] or decrease [16] under low light intensity conditions, depending on the type of plant. Low light intensity, for example, increased methylxanthine-content in the leaves of *Ilex paraguariensis* [17], but decreased resin content in the leaves of *Grindelia chiloensis* [14].

Previous studies have shown that differences in irradiance levels were able to change the production of secondary metabolites in plants [18,19,20]. This simultaneously can affect the medicinal components in these plants, besides changes in the morphology and physiology [21,22]. Both Briskin [21] and Kurata [22] observed a positive and significant relationship between production of total phenolics and flavonoids, and antioxidant activities in plants. It seems that different irradiances had a direct effect on antioxidant activities in plants resulting in increased total phenolics and flavonoids contents. It was suggested that increasing phenolic and flavonoid components in shaded plants are related to lower temperatures under this light condition. High temperature increases anthocyanin degradation in grape skin, together with a decrease in expression of flavonoids biosynthesis [23]. On the other hand, low temperature increases anthocyanin production, which previously has been observed in grape berries [24,25]. 

It has been shown that plants irradiated with high light intensity tend to increase their photosynthetic capacity and enhance the biomass [26]. Many studies have investigated the effects of high light levels on the vegetative and plant primary metabolism but relatively few studies have investigated the response of plant carbon based secondary metabolites (CBSM) to decreasing irradiance [27], particularly in a herbal plant such as *O. stimaneus*. Previous research by Affendy *et al*. [28] have shown that *O. stimaneus* are sensitive to low light intensity however, the result was not conclusive because it only measured growth parameters. The objective of the current study was to examine the effects of different irradiance on secondary metabolites (flavonoids and phenolics), H_2_O_2_ and malondialdehyde (MDA) in *O. stimaneus*, and to determine how secondary metabolites were related to primary (total non-structural carbohydrate) metabolites, C/N ratio, chlorophyll content, and photosynthesis rate. These findings would be a useful tool in determining plant growth requirements for quality enhancement of medicinal plants, especially *O. stimaneus*.

## 2. Results and Discussion

### 2.1. Total Phenolics and Total Flavonoids Profiling

Different irradiance levels had a significant (*P *≤ 0.05) impact on the production of total phenolics and flavonoids (Table 1). As the light intensity increased from 225 µmol/m^2^/s to 900 µmol/m^2^/s, the *O. stimaneus* plants produced less total phenolics and flavonoids. 

**Table 1:** Accumulation and partitioning of total flavonoids (TF) and total phenolics (TP) in different plant parts of *Orthosiphon stimaneus *under different irradiance levels.

Irradiance (µmol/m^2^/s)	Plant Parts	Total flavonoids, TF (mg rutin/g dry weight)	Total phenolics, TP (mg gallic acid/g dry weight)
	Leaf	2.111 ± 0.013 ^a^	5.211 ± 0.028 ^a^
225	Stem	1.991 ± 0.022 ^a^	4.811 ± 0.029 ^a^
	Root	1.671 ± 0.013 ^b^	4.671 ± 0.039 ^b^
	Leaf	1.567 ± 0.022 ^b^	4.211 ± 0.032 ^b^
500	Stem	1.321 ± 0.030 ^b^	3.981 ± 0.037 ^b^
	Root	1.231 ± 0.022 ^c^	3.761 ± 0.051 ^c^
	Leaf	1.234 ± 0.013 ^c^	3.111± 0.021 ^c^
675	Stem	1.001 ± 0.010 ^c^	2.981 ± 0.025 ^c^
	Root	0.987 ± 0.015 ^d^	2.761 ± 0.040 ^c^
	Leaf	0.913 ± 0.025 ^d^	2.345± 0.008 ^d^
900	Stem	0.813 ± 0.023 ^d^	1.981 ± 0.011 ^d^
	Root	0.723 ± 0.026 ^d^	1.721 ± 0.028 ^d^

All analyses are mean ± standard error of mean (SEM). N = 40. Means not sharing a common letter were significantly different at P ≤ 0.05.

The accumulation of secondary metabolites was also found to be more pronounced in the leaves, followed by the stems and the roots. As the irradiance decreased from 900 to 675 and then 500 µmol/m^2^/s the foliar total flavonoids decreased subsequently by 56, 41 and 26%, respectively, compared to irradiance at 225 µmol/m^2^/s. A similar decreasing trend was also observed for foliar total phenolics with plants being exposed to 900 µmol/m^2^/s registering the lowest value of total phenolics production (2.35 mg gallic acid/g dry weight), compared to 225 µmol/m^2^/s irradiance, which recorded 5.21 mg gallic acid/g dry weight. The increase in total plant flavonoids and total phenolics under low irradiance was also reported in previous studies by Jaafar *et al*. [19], Wand [29] and Liakura *et al*. [30].

The increase in plant secondary metabolites under low irradiance might be due to an increase in the production of total non-structural carbohydrates (TNC), as exhibited by the high correlation coefficient (R^2^ = 0.97; flavonoids and R^2^ = 0.96; phenolics; P ≤ 0.05) in Table 2. It was observed that, the increase in sucrose content might have more influence on the up regulation of secondary metabolites, compared to starch content, which registered lower correlation coefficients with both the total flavonoids (R^2^ = 0.76 *vs.* R2 = 0.87; P ≤ 0.05) and phenolics (R^2^ = 0.81 *vs.* 0.89; P ≤ 0.05). The data implies that increase in sucrose content in *O. stimaneus* might be a possible explanation of increase in production of total flavonoids, and phenolics compared to starch increase in the present study. The result was in agreement with the finding of Guo *et al*. [31], who proposed that the increase in production of plant secondary metabolites observed in their studies on broccoli was due to increased production of sucrose. Meanwhile, Tsormpatsidis *et al*. [32] proposed that the increase in carbon based secondary metabolites production (total phenolics and flavonoids) under low irradiance was due to increased availability of phenyl alanine, which is the precursor for CBSM, that justifies increased production of these compounds under low light condition. These results entailed that the production of plant secondary metabolites in *O. stimanues* was favored under low irradiance levels. The increase in production of secondary metabolites under low light levels was also observed in tobacco plant [33]. The present result was also in agreement with resource allocation hypothesis that was proposed by Coley *et al*. [34] who hypothesized the up-regulation of secondary metabolites production under low light condition.

### 2.2. Total Soluble Sugar, Starch and Total Non Structural Carbohydrate (TNC) and Their Profiling

The profiling of carbohydrates was influenced by the irradiance levels applied to *O. stimaneus *(P ≤ 0.01). The accumulation of carbohydrates in different parts of the plant followed a descending order of leaf > stem > root. As irradiance levels decreased, the concentration of sucrose, starch and TNC increased (Table 3). It was found that, the concentration of sucrose and starch registered the lowest values in 900 µmol/m^2^/s irradiance compared to other treatments. In the leaves, the 900 µmol/m^2^/s (22.80 mg sucrose/g dry weight), 675 µmol/m^2^/s (28.64 mg sucrose/g dry weight) and 500 µmol/m^2^/s (33.45 mg sucrose/g dry weight) irradiance treatments produced less sucrose than the 225 µmol/m^2^/s treatment which produced 45.71 mg sucrose/g dry weight.

**Table 2:** Correlations among the measured parameters in the experiments.

Parameters	1	2	3	4	5	6	7	8	9	10	11	12	13
**1. Flavonoid**	1.00												
**2. Phenolics**	0.89 *	1.00											
**3. Sucrose**	0.87 *	0.89 *	1.00										
**4. Starch**	0.76 *	0.81 *	0.89 *	1.00									
**5. TNC**	0.97 *	0.96 *	0.91 *	0.78 *	1.00								
**6. H_2_O_2_**	0.88 *	0.92 *	0.76 *	0.88 *	0.79 *	1.00							
**7. MDA**	0.87 *	0.87 *	0.91 *	0.96 *	0.9 *	0.86 *	1.00						
**8. Photo**	−0.87 **	−0.86 *	0.56	−0.90 *	−0.56	−0.78	−0.87	1.00					
**9. C/N**	0.79 *	0.88 *	0.78	0.87 *	0.68 *	0.74 *	0.76	−0.84	1.00				
**10. Biomass**	−0.87 *	−0.78 *	−0.89 *	−0.67	−0.68 *	−0.58	−0.54	−0.66	−0.54	1.00			
**11. Chl a**	−0.78 *	−0.69	−0.67	−0.78 *	−0.57	−0.45	−0.67 *	−0.54	−0.44	−0.67	1.00		
**12. Chl b**	−0.56	−0.75 *	−0.76 *	−0.75 *	−0.67 *	−0.54	−0.71 *	−0.45	−0.18	−0.65	0.67 **	1.00	
**13.T. Chl**	−0.78 *	−0.71 *	−0.81 *	−0.71 *	−0.53	−0.51	−0.69 *	−0.58 *	−0.34	0.67 *	0.68 *	0.68 *	1.00

* and ** respectively significant at *P* ≤ 0.05 or *P* ≤ 0.01. TNC, total non-structurable carbohydrate; MDA, maliondiadehyde; Photo, net photosynthesis; Chl a, chlorophyll a; Chl b, chlorophyll b; T. Chl, total chlorophyll.

The starch content in the leaves at 900 µmol/m^2^/s was statistically lower than other irradiance treatments. In the experiment, the foliar starch contents at 225, 500, 625 µmol/m^2^/s irradiance treatments were 96.41, 79.18 and 61.22 mg glucose/g dry weight, respectively, compared to only 46.66 mg glucose/g dry weight at 900 µmol/m^2^/s irradiance. In all plant parts of *O. stimaneus*, the increase in starch content was larger than the increase in sugar concentration [35]. Results suggested that low irradiance was able to enhance the soluble sugar and starch contents, which previously had simultaneously enhanced the TNC. Shure and Wilson [36] have attributed the increase in plant polyphenols production under low light to increase in production of starch and soluble sugar. 

**Table 3:** Accumulation and partitioning of soluble sugar, starch and total non structurable carbohydrate (TNC) in different plant parts of *Orthosiphon stimaneus *under different irradiance levels.

Irradiance (µmol/m^2^/s)	Plant parts	Soluble sugar (mg sucrose/g dry weight)	Starch (mg glucose/g dry weight)	TNC (mg/g dry weight)
	Leaf	45.71 ± 0.50 ^a^	96.41 ± 0.68 ^a^	141.32 ± 3.22 ^a^
225	Stem	33.04 ± 0.84 ^b^	88.26 ± 0.19 ^a^	121.21 ± 1.46 ^a^
	Root	32.09 ± 0.50 ^b^	81.22 ± 0.59 ^b^	113.34 ± 3.26 ^b^
	Leaf	33.45 ± 0.49 ^b^	79.18 ± 0.52 ^b^	112.17 ± 4.77 ^b^
500	Stem	31.26 ± 0.44 ^b^	68.27 ± 0.37 ^c^	100.23 ± 2.22 ^c^
	Root	29.66 ± 0.49 ^c^	62.98 ± 0.11 ^c^	91.21 ± 5.55 ^c^
	Leaf	28.64 ± 0.59 ^c^	61.22 ± 0.13 ^c^	89.11 ± 4.67 ^c^
675	Stem	27.10 ± 0.99 ^c^	57.03 ± 0.15 ^d^	84.21 ± 4.90 ^c^
	Root	24.86 ± 0.58 ^c^	51.67 ± 0.44 ^d^	75.21 ± 1.90 ^d^
	Leaf	22.80 ± 1.16 ^d^	46.66 ± 0.23 ^d^	68.12 ± 5.89 ^d^
900	Stem	14.96 ± 0.70 ^d^	37.42 ± 0.21 ^d^	52.12 ± 1.65 ^e^
	Root	12.02 ± 1.17 ^d^	31.20 ± 0.28 ^e^	43.12 ± 9.96 ^e^

All analyses are mean ± standard error of mean (SEM), N = 40. Means not sharing a common single letter were significantly different at P ≤ 0.05.

Total structural carbohydrates are the basic compounds required to produce carbon-based secondary metabolites in the shikimic acid pathway [37]. Ibrahim and Jaafar [35] found that increase in the production of total phenolics and flavonoids in herbal medicinal plant *Labisia pumila* was due to an increase in the production of TNC. In the present study, the increase in the production of TNC was also found to establish a good positive correlation with secondary metabolites as depicted by the significant (P ≤ 0.05) correlation coefficients in Table 2 with total flvonoids (R^2^ = 0.97; P ≤ 0.05) and total phenolics (R^2^ = 0.96; P ≤ 0.05). Katsube *et al*. [38] proposed that the increase in phenolics and flavonoids production was related to the balance between carbohydrate source and sink, the greater the source-to-sink ratio, the greater would production of plant secondary metabolites be. A similar trend of increasing total phenolics and flavonoids with increasing production of TNC under low light levels was also observed in *Hypericum perfotum* and other medicinal plants [39,40].

### 2.3. H_2_O_2_ and Lipid Peroxidation Activity

The production of hydrogen peroxide (H_2_O_2_) and malondialdehyde (MDA) was influenced by irradiance levels imposed onto *O. stimaneus* seedlings (P ≤ 0.01). It was observed that as the levels of irradiance decreased from 900 to 225 µmol/m^2^/s the production of H_2_O_2_ and MDA was also reduced (Table 4). At all irradiance levels, the highest contents of H_2_O_2_ and MDA were noted in leaves, followed by the stems, and the lowest in the roots. From the correlation Table 2, the H_2_O_2_ and MDA production have established significant positive correlations with total flavonoids and total phenolics, indicating that increase in H_2_O_2_ and MDA might be involved in the up-regulation of the secondary metabolites production under low light condition in *O. stimaneus*. 

**Table 4:** Accumulation and partitioning of H_2_O_2_ and MDA in different plant parts of *Orthosiphon stimaneus *under different irradiance levels.

Irradiance (µmol/m^2^/s)	Plant Parts	H_2_O_2_ (ng/g fresh weight)	MDA (µmol/g fresh weight)
	Leaf	0.15 ± 0.08 ^a^	2.11 ± 0.09 ^a^
225	Stem	0.09 ± 0.01 ^a^	1.82 ± 0.12 ^a^
	Root	0.07 ± 0.01 ^b^	0.98 ± 0.13 ^c^
	Leaf	0.11 ± 0.04 ^a^	1.98 ± 0.24 ^a^
500	Stem	0.06 ± 0.06 ^b^	1.67 ± 0.21 ^b^
	Root	0.04 ± 0.06 ^c^	0.87 ± 0.02 ^c^
	Leaf	0.09 ± 0.03 ^a^	1.87 ± 0.13 ^a^
675	Stem	0.04 ± 0.01 ^c^	1.43 ± 0.07 ^b^
	Root	0.02 ± 0.04 ^d^	0.77 ± 0.04 ^d^
	Leaf	0.08 ± 0.02 ^b^	1.26 ± 0.02 ^b^
900	Stem	0.03 ± 0.01 ^c^	1.01 ± 0.03 ^c^
	Root	0.01 ± 0.01 ^d^	0.68 ± 0.01 ^d^

All analyses are mean ± standard error of mean (SEM), N = 40. Means not sharing a common single letter were significantly different at P ≤ 0.05.

The production of reactive oxygen species (ROS) such as H_2_O_2_ in plants, also known as oxidative burst, is an early event of plant response to different stress level, and acts as a signal to the production of secondary metabolism [41]. The formation of H_2_O_2_ is the most important phenomenon of signal transduction induced by stress conditions which change the content of redox agents and the redox state of cells [42]. The increase in H_2_O_2_ content with corresponding increase in total flavonoids and phenolics was observed by Jabs *et al*. [43]. The formation of malondialdehyde (MDA) was considered as a measure of lipid peroxidation that was induced by low irradiance levels. MDA, a decomposition product of polyunsaturated fatty acids hydroperoxides, has been utilized very often as a suitable biomarker for oxidative stress. Total MDA activity documented in low irradiance (225 µmol/m^2^/s) was significantly higher in the ambient (900 µmol/m^2^/s) indicating higher stress levels exhibited by plants under low irradiance condition. These results suggested the occurrence of lipid peroxidation in *O. stimaneus* under low irradiance probably as a consequence of higher amount of H_2_O_2_ levels. Hydrogen peroxide may function as a signal for the induction of plant defence systems and this could enhance secondary metabolite production [44].Thus, it could be speculated that a cascade of events including lipid peroxidation and accumulation of H_2_O_2_ content might be involved in the initiation of secondary metabolite production. It also appeared that the oxidative stress is a pre-requisite for secondary metabolite synthesis in *O. stimaneus* under low irradiance level.

### 2.4. Net Photosynthesis

The photosynthesis rate was influenced by the irradiance levels. As irradiance levels increased in an ascending order 225 µmol/m^2^/s > 500 µmol/m^2^/s > 625 µmol/m^2^/s > 900 µmol/m^2^/s the photosynthesis rate also increased steadily (Table 5). The highest photosynthesis in *O. stimaneus *was obtained under 900 µmol/m^2^/s (11.71 µmol/m^2^/s) followed by 625 µmol/m^2^/s (9.31 µmol/m^2^/s), 500 µmol/m^2^/s (6.21 µmol/m^2^/s), and the lowest under 225 µmol/m^2^/s (2.01 µmol/m^2^/s) irradiance. The increase in photosynthetic rate under high irradiance was attributed to increase in stomatal conductance, transpiration rate, stomatal index and stomatal density under this condition [45,46]. The result from the current study indicated that the production of secondary metabolites was up-regulated when photosynthesis decreased. This was shown by the significant (P ≤ 0.01) negative correlations of net photosynthesis with total phenolics (R^2^ = −0.86) and total flavonoids (R^2^ = −0.87) in Table 2. A possible explanation for this outcome is that the decreasing photosynthetic rate in this study might have up-regulated the shikimic acid and pentose phosphate pathway activities [43]. An increase in shikimic acid and pentose phosphate pathway under low photosynthesis rate was observed by Fan [47]. Ibrahim *et al*. [48] also reached a similar conclusion with *Labisia pumila* when they observed that the up-regulation of secondary metabolite production was related to a reduction in net photosynthesis due to accumulation of TNC in the leaf. In the correlation table, it is also seen that net photostynthesis has a significant (P ≤ 0.01) negative relationship with starch (R^2^ = 0.90), suggesting that reduction in net photosynthesis under low light levels might be due to accumulation of starch in the leaf that is usually related to an impairment of net photosynthesis [35].

**Table 5:** The effects of different irradiance levels on net photosynthesis, leaf nitrogen, C/N ratio and total chlorophyll content in *Orthosiphon stimaneus.*

Parameters	Irradiance (µmol/m^2^/s)			
	225	500	675	900
Photosynthesis (µmol/m^2^/s)	4.01 ± 0.25 ^d^	6.21 ± 0.34 ^c^	9.31 ± 0.12 ^b^	11.71 ± 0.43 ^a^
C/N ratio	20.35 ± 1.21 ^a^	17.73 ± 0.02 ^b^	14.79 ± 0.05 ^c^	10.12 ± 0.23 ^d^
Total biomass (g)	25.21± 0.12 ^d^	27.21 ± 0.02 ^c^	32.79 ± 0.23 ^b^	35.26 ± 0.43 ^a^
Chlorophyll a (mg/g fresh weight)	3.61 ± 0.01 ^d^	4.22 ± 0.04 ^c^	5.33 ± 0.09 ^b^	6.77 ± 0.08 ^a^
Chlorophyll b (mg/g fresh weight)	17.27 ± 0.23 ^d^	18.23 ± 0.09 ^c^	24.72 ± 0.34 ^b^	28.76 ± 0.23 ^a^
Total chlorophyll (mg/g fresh weight)	20.13 ± 0.11 ^d^	22.14 ± 0.45 ^c^	29.78 ± 0.89 ^b^	34.23 ± 0.98 ^a^

All analyses are mean ± standard error of mean (SEM). N = 40. Means not sharing a common single letter are significantly different at P ≤ 0.05.

### 2.5. C/N Ratio

The increment of irradiance significantly reduced the C/N ratio (P ≤ 0.05). As irradiance levels increased from 225 to 900 µmol/m^2^/s the foliar C/N ration declined considerably. The C/N ratio at 225 µmol/m^2^/s was 12%, 27% and 52% higher than 500, 625 and 900 µmol/m^2^/s irradiance treatments, respectively. High C/N ratio had a significant positive relationship (P ≤ 0.01) with total flavonoids (R^2^ = 0.79; P ≤ 0.05) and total phenolic compounds (R^2^ = 0.88; P ≤ 0.05; Table 2) signifying a good direct association between the C/N ratio and plant secondary metabolites. Conversely, the C/N ratio displayed a significant negative relationship with photosynthesis (R^2^ = −0.84; P ≤ 0.05), implying that increase in C/N ratio decreased the photosynthetic capacity of *O. stimaneus*. Winger *et al*. [49] attributed the increase in C/N ratio with decreasing photosynthetic capacity to an increase in carbohydrate accumulation, which repressed photosynthetic protein production, especially Rubisco. In the present study, the increase in C/N ratio had also reduced the photosynthetic capacity of *O. stimaneus *seedlings, and this suggested an enhanced synthesis of plant secondary metabolites, especially flavonoids and phenolics [50]. The high C/N ratio of *O. stimanues* under low light also indicates a reduced nitrogen content condition in the leaves. Generally, plants under low nitrogen supply have low sink strength that signifies increase in production of TNC which up regulates the production of total phenolics and flavonoids due to reducing translocation of carbohydrates to other plant parts [51]. Cronin and Lodge [52] reported that under high irradiance the leaf nitrogen decreased substantially, which increased the C/N ratio and justified the increase in polyphenolics production.

### 2.6. Total Plant Biomass

Different irradiance levels significantly (P ≤ 0.01) affected plant biomass production. With decreasing light levels, plant biomass decreased significantly. The highest production of plant biomass (35.26 g) was observed at 900 µmol/m^2^/s, followed by 625 µmol/m^2^/s (32.79 g), 500 µmol/m^2^/s (27.21 g) and 225 µmol/m^2^/s (25.21 g) irradiance. The reduced total plant biomass under low irradiance might be due to a decrease in photosynthetic rate [53]. At the same time, total biomass demonstrated a significant negative relationship with the production of secondary metabolites (Table 2) suggesting that the decrease in biomass accumulation was related to increase in the concentration of total phenolics and flavonoids. This result indicates that there was a trade-off between growth and secondary metabolism under low light conditions [17]. Afreen *et al*. [54] regarded low light intensity as an environmental stimulus for the production of secondary metabolites in some herb plants. This implies that under low irradiance levels secondary metabolites production of *O. stimaneus* was up-regulated while the biomass production was reduced.

### 2.7. Chlorophyll Content

Chlorophyll content was influenced by light intensity (*P *≤ 0.01). As the levels of irradiance increased from 225 to 900 µmol/m^2^/s, chlorophyll a, b and total chlorophyll a+b were also enhanced. The increase in chlorophyll content with increasing irradiance has been reported by Suza *et al*. [55]. It was found from the correlation analyses (Table 2) that chlorophyll a, b and total chlorophyll were significantly (P ≤ 0.01) and negatively related with secondary metabolites. Competition between secondary metabolites and chlorophyll contents fits well with the prediction of protein competition model (PCM) where secondary metabolites content is controlled by the competition between protein and secondary metabolites biosynthesis pathway and its metabolites regulation. The negative relationship between secondary metabolites and chlorophyll content is a sign of gradual switch of investment from protein to polyphenolics production [56]. Results from the present study indicate that the production of chlorophyll content competes with the production of secondary metabolites. In the present study, it was also found that under low light levels nitrogen was significantly reduced as demonstrated by high C/N ratio. The increase in the production of secondary metabolites of *O. stimaneus* under low irradiance might be due to increase in availability of phenyl alanine (phe), a precursor for secondary metabolites and protein production, where the production of secondary metabolites was more prioritized under low nitrogen levels due to the restriction of protein production as exhibited by reduced chlorophyll production in the current study [57].

## 3. Experimental

### 3.1. Plant Material and Maintenance

The experiment was carried out in glasshouses at the Faculty of Agriculture Glasshouse Complex, Universiti Putra Malaysia (longitude 101°44’N and latitude 2°58’S, 68 m above sea level) with a mean atmospheric pressure of 1.013 kPa. Stem cuttings of *O. stimanues* was propagated for two weeks in small pots and then transferred to white polyethylene bags filled with a soilless mixture of burnt rice husk and coco peat (ratio 3:1). *O. stimaneus* is a semi-shade plant that requires some amount of shade for maximum production. In order to determine the shade level for maximum production, the plants were grown under four levels of glasshouse shade, *viz*. 0% (control of no shading), 20%, 40% and 60% shade levels, which were equivalent to average light intensity passing through each shading treatment of 900, 675, 500, and 225 µmol/m^2^/s, respectively. The experiment was based on a Randomized Complete Block Design (RCBD) with four replicates. Each treatment consisted of 10 plants totaling a sum of 160 plants in the experiment. Plants were harvested at 12 weeks after planting.

### 3.2. Standard and Reagents

Standard of gallic acid, rutin, sucrose, glucose, MDA and H_2_O_2_ were purchased from Sigma-Aldrich (USA). The standard reagent in the chemical analysis of Folin-Ciocalteu, Anthrone, potassium iodide (KI), trichloroacetic acid (TCA), thiobarbituric acid (TBA) and K-phosphate buffer were obtained from Fisher Chemical (UK).

### 3.3. Total Phenolics and Total Flavonoids Quantification

The method of extraction and quantification for total phenolics and flavonoids contents followed after Ibrahim and Jaafar [58]. An amount of ground (0.25 mm) tissue samples (0.1 g) was extracted with 80% ethanol (10 mL) on an orbital shaker for 120 minutes at 50 °C. The mixture was subsequently filtered (Whatman™ No. 1), and the filtrate was used for the quantification of total phenolics and total flavonoids. Folin-Ciocalteu reagent (diluted 10-fold) was used to determine the total phenolics content of the leaf samples. Two hundred µL of the sample extract was mixed with Follin-Ciocalteau reagent (1.5 mL) and allowed to stand at 22 °C for 5 minutes before adding NaNO_3_ solution (1.5 mL, 60 g L^−1^). After two hours at 22 °C, absorbance was measured at 725 nm. The results were expressed as mg g^−1^ gallic acid equivalent (mg GAE g^−1^ dry sample). For total flavonoids determination, a sample (1 mL) was mixed with NaNO_3_ (0.3 mL) in a test tube covered with aluminum foil, and left for 5 minutes. Then 10% AlCl_3_ (0.3 mL) was added followed by addition of 1 M NaOH (2 mL). Later, the absorbance was measured at 510 nm using a spectrophotometer with rutin as a standard (results expressed as mg g^−1^ rutin dry sample).

### 3.4. Sucrose Determination

Sucrose was measured spectrophotometrically using the method of Ibrahim and Jaafar [59]. Samples (0.5 g; 0.25 mm) were placed in 15 mL conical tubes, and distilled water added to make up the volume to 10 mL. The mixture was then vortexed and later incubated for 10 minutes. Anthrone reagent was prepared using anthrone (0.1 g) that was dissolved in 95% sulphuric acid (Fisher Scientific, USA, 50 mL). Sucrose was used as a standard stock solution to prepare a standard curve for the quantification of sucrose in the sample. The mixed sample of ground dry sample and distilled water was centrifuged at a speed of 3,400 rpm for 10 minutes and then filtered to get the supernatant. A sample (4 mL) was mixed with anthrone reagent (8 mL) and then placed in a water-bath set at 100 °C for 5 minutes before the sample was measured at an absorbance of 620 nm using a spectrophotometer model UV160U (Shimadzu Scientific, Kyoto, Japan). The soluble sugar in the sample was expressed as mg sucrose g^−1^ dry sample.

### 3.5. Starch Determination

Starch content was determined spectrophometrically using a method described by Thayumanavam and Sadasivam [60]. In this method, ground (0.25 mm) dry sample (0.5 g) was homogenized in hot 80% ethanol to remove the sugar. The sample was then centrifuged at 5,000 rpm for 5 minutes and the residue retained. After that, distilled water (5.0 mL) and 52% perchloric acid (6.5 mL) were added to the residue. Then the solution was centrifuged and the supernatant separated and then filtered with Whatman No. 5 filter paper. The processes were repeated until the supernatant was made up to 100 mL. A sample (100 µL) of the supernatant was added to distilled water in a test tube until the volume became 1 mL. After that, anthrone reagent (4 mL, prepared with 95% sulphuric acid) was added to the test tube. The mixed solution was placed in the water bath at 100 °C for eight minutes and then cooled to room temperature, and then the sample was read at absorbance of 630 nm to determine the sample starch content. Glucose was used as a standard and starch content was expressed as mg glucose equivalent g^−1^ dry sample.

### 3.6. Total Soluble Sugar and Total Non Structural Carbohydrate (TNC)

The total non structural carbohydrate was calculated as the sum of total soluble sugar and starch content [61].

### 3.7. Measurement of H_2_O_2_ and Malondialdehyde (MDA) Content

Hydrogen peroxide content of the plant parts was measured spectrophotometrically after reaction with potassium iodide (KI) [62]. The reaction mixture consisted of 0.1% trichloroacetic acid (TCA, 0.5 mL), plant leaf, stem and root extract supernatant (0.25 mm), 100 mM K-phosphate buffer (0.5 mL) and reagent (2 mL, 1 M KI, w/v in fresh double-distilled water). The blank probe consisted of 0.1% TCA in the absence of plant extract. After 1 hour of reaction in darkness, the absorbance was measured at 390 nm. The amount of hydrogen peroxide was calculated using a standard curve prepared with known concentrations of H_2_O_2_. Lipid peroxidation of plant parts was estimated by the level of malondialdehyde (MDA) production using thiobarbituric acid (TBA) method as described by Heath and Packer [63]. One gram of ground (0.25 mm) plant sample was homogenized with a mortar and pestle in 0.5% trichloracetic acid (TCA, 1 mL). The homogenate was centrifuged at 9,000 rpm for 20 min. The supernatant (0.5 mL) was mixed with 20% TCA (2.5 mL) containing 0.5% TBA and heated in a boiling water bath for 30 min and allowed to cool in an ice bath quickly. The supernatant was centrifuged at 9,000 rpm for 10 min, and resulting supernatant was used for determination of MDA content. Absorbance at 532 nm was recorded.

### 3.8. Photosynthesis Rate

The measurement was obtained from a closed infra-red gas analyzer LICOR 6400 Portable Photosynthesis System (IRGA, Licor Inc., USA). Prior to use, the instrument was warmed for 30 minutes and calibrated with the ZERO IRGA mode. Two steps are required in the calibration process: first, the initial zeroing process for the built-in flow meter; and second, zeroing process for the infra-red gas analyzer. The measurements used optimal conditions set by Ibrahim *et al*. [64] of 400 µmol mol^−1^ CO_2_ 30 °C cuvette temperature, 60% relative humidity with air flow rate set at 500 cm^3^ min^−1^, and modified cuvette condition of 800 µmol m^−2^ s^−1^ photosynthetically photon flux density (PPFD). The measurements of gas exchange were carried out between 09:00 to 11 :00 a.m. using fully expanded young leaves to record net photosynthesis rate (A). The operation was automatic and the data were stored in the LI-6400 console and analyzed by “ *Photosyn Assistant *“software (Version 3, Lincoln Inc., USA). Several precautions were taken to avoid errors during measurements. Leaf surfaces were cleaned and dried using tissue paper before enclosed in the leaf cuvette.

### 3.9. Plant Biomass

Total plant biomass was taken by calculating the dry weight of root, stem and leaf per seedling. Plant parts were separated and placed in paper bags and oven dried at 80 °C until constant weight was reached before dry weights were recorded using electronic weighing scale (Mettler-Toledo Model B303-S, Switzerland). 

### 3.10. Total Carbon, Nitrogen and C:N Ratio

Total carbon and C:N ratio were measured by using a CNS 2000 analyzer (Model A Analyst 300, LECO Inc., USA). This was performed by placing ground (0.25 mm) leaf sample (0.05 g) into the combustion boat. Successively, the combustion boat was transferred to the loader before the sample was burned at 1,350 °C to obtain the reading of total carbon and nitrogen content of the samples [65,66,67].

### 3.11. Chlorophyll Content

Total chlorophyll content was measured by method from Idso *et al*. [68] using fresh weight basis. Prior to destructive harvest each seedling was analyzed for the leaf relative chlorophyll reading (SPAD meter 502, Minolta Inc., USA). The leaves of *O. stimaneus* with different greenness (yellowish green, light green and dark green) were selected for analysis and total leaf chlorophyll content was analyzed. For each type of leaf greenness, the relative SPAD value was recorded (five points/leaf) and the same leaves sampled for destructive chlorophyll content determination in which leaf disk of 3 mm in diameter was obtained from leaf sample using a hole puncher. For each seedling the measurement was conducted on the youngest fully expanded leaves of each plant, generally the second or third leaf from the tip of the stem was used. The leaf disks were immediately immersed in acetone (20 mL) in an aluminum foil-covered glass bottle for approximately 24 hours at 0 °C until all the green colour had bleached out. Finally, the solution (3.5 mL) was transferred to measure at absorbances of 664 and 647 nm using a spectrometer (UV-3101P, Labomed Inc, USA). After that the least squares regression was used to develop predictive relation between SPAD meter readings and pigment concentrations (mg g^−1^ fresh weight) obtained from the chlorophyll destructive analysis.

### 3.12. Statistical Analysis

Data were analyzed using analysis of variance by SAS version 17. Mean separation test between treatments was performed using Duncan multiple range test and standard error of differences between means was calculated with the assumption that data were normally distributed and equally replicated.

## 4. Conclusions

The current study has demonstrated that exposure to high irradiance can reduce the production of total flavonoids and phenolics in *O. stimaneus*. The increased production of carbon-based secondary metabolites (CBSM) under low irradiance was also followed by enhancement in the production of total non-structural carbohydrate (TNC), H_2_O_2_ and MDA, which were related to the increased secondary metabolites production. The low irradiated plants exhibited a negative relationship with total chlorophyll content and registered high C/N ratios. As the irradiance was reduced, net photosynthesis and total biomass also decreased, indicating the possible existence of a trade-off between growth and production of secondary metabolites in low irradiated plants, confirming the resource availability hypothesis that the production of plant secondary metabolites might be up-regulated under low irradiance levels

## References

[1] Wangner H. (1982). Parmazietische Biologie: Drogen und ihre Inhaltsstoffe.

[2] Olah N.K., Radu L., Mogosan C., Hanganu D., Gocan S. (2003). Phytochemical and pharmacological studies on *Orthosiphon stamineus* Benth. (Lamiaceae) hydroalcoholic extracts. J. Pharm. Biomed. Anal..

[3] Tezuka Y., Stampoulis P., Banskota A.H., Awale S., Tran K.Q., Saiki I., Kadota S. (2000). Constituents of the Vietnamese medicinal plant *Orthosiphon stamineus*. Chem. Pharm. Bull..

[4] Hollman P.C., Katan M.B. (1999). Dietary flavonoids: Intake, health effects and bioavailability. Food Chem. Toxicol..

[5] Lyckander I.M., Malterud K.E. (1996). Lipophilic flavonoids from *Orthosiphon spicatus* prevent oxidative inactivation of 15-lipoxygenase. Prostaglandins Leukot. Essent. Fatty Acids.

[6] Schut G.A., Zwaving J.H. (1993). Pharmacological investigation of some lipophilic flavonoids from *Orthosiphon aristatus*. Fitoterapia.

[7] Hollman P.C., Katan M.B. (1999). Dietary flavonoids; intake, health effects and bioavailability. Food Chem. Toxicol..

[8] Rice-Evans C.A., Packer L. (1998). Flavonoids in Health and Disease.

[9] Fine P.V.A., Miller Z.J., Mesones I., Irazuzta S., Appel H.M., Stevens M.H.H. (2006). The growth defense trade off and habitat specialization by plants in Amazonian forest. Ecology.

[10] Hemm M.R., Rider S.D., Ogas J., Murry D.J., Chapple C. (2004). Light induces phenylpropanoid metabolism in *Arabidopsis* roots. Plant J..

[11] Liu C.Z., Guo C., Wang Y.C., Ouyang F. (2002). Effect of light irradiation on hairy root growth and artemisinin biosynthesis of Artemisia annua. Process Biochem..

[12] Xie B.D., Wang H.T. (2006). Effects of light spectrum and photoperiod on contents of flavonoid and terpene in leaves of *Ginkgo biloba* L. Nanjing For. Univ..

[13] Bryant J.P., Chapin F.S., Klein D.R. (1983). Carbon nutrient balance of boreal plants in relation to vertebrate herbivory. Oikos.

[14] Zavala J.A., Ravetta D.A. (2001). Allocation of photoassimilates to biomass, resin and carbohydrates in *Grindelia chiloensis* as affected by light intensity. Field Crops Res..

[15] Chauser-Volfson E., Gutterman Y. (1998). Content and distribution of anthrone C-glycosides in the South African arid plant species *Aloe mutabilis* growing in the direct sunlight and the shade in the Negev Desert of Israel. J. Arid Environ..

[16] Gershenzon J. (1994). Metabolic cost of terpenoid accumulation in higher plants. J. Chem. Ecol..

[17] Coelho G.C., Rachwal M.F.G., Dedecek R.A., Curcio G.R., Nietsche K., Schenkel E.P. (2007). Effect of light intensity on methylxanthine contents of *Ilex paraguariensis* A. St. Hil. Biochem. Syst. Ecol..

[18] Graham T.L. (1998). Flavonoid and flavonol glycoside metabolism in *Arabidopsis*. Plant Physiol. Biochem..

[19] Jaafar H.Z., Mohamed Haris N.B., Rahmat A. (2008). Accumulation and partitioning of total phenols in two varieties of *Labisia pumila* Benth. under manipulation of greenhouse irradiance. Acta Hortic..

[20] Gu X.D., Sun M.Y., Zhang L., Fu H.W., Cui L., Chen R.Z., Zhang D.W., Tian J.K. (2010). UV-B induced changes in the secondary metabolites of *Morus alba *L. Leaves. Molecules.

[21] Briskin D.P., Gawienowski M.C. (2001). Differential effects of light and nitrogen on production of hypericins and leaf glands in *Hypericum perforatum*. Plant Physiol..

[22] Kurata H., Matsumura S., Furusaki S. (1997). Light irradiation causes physiological andmetabolic changes for purine alkaloid production by a *Coffea arabica* cell suspension culture. Plant Sci..

[23] Mori K., Goto-Yamamoto N., Kitayama M., Hashizume K. (2007). Loss of anthocyanins in red-wine grape under high temperatura. Exp. Bot..

[24] Mori K., Sugaya S., Gemma H. (2005). Decreased anthocyanin biosynthesis in grape berries grown under elevated night temperature condition. Sci. Hortic..

[25] Yamane T., Jeong S.T., Goto-Yamamoto N., Koshita Y., Kobayashi S. (2006). Effects of temperature on anthocyanin biosynthesis in grape berry skins. Am. J. Enol. Vitic..

[26] Abrams M.D., Mostoller S.A. (1995). Gas exchange, leaf structure and nitrogen in contrasting successional tree species growing in open and understory sites during a drought. Tree Physiol..

[27] Awale S., Tezuka Y., Banskota A.H., Kouda K., Tun K.M., Kadota S. (2001). Five novel highly oxygenated diterpenes of *Orthosiphon stamineus* from Myanmar. J. Nat. Prod..

[28] Affendy H., Aminuddin M., Azmy M., Amini M.A., Assis K., Tamir A.T. (2010). Effects of light intensity on *Orthosiphon stamineus* Benth treated with different organic fertilizers. Int. J. Agric. Res..

[29] Wand S.J.E. (1995). Concentration of ultraviolet-B radiation absorbing compounds in leaves of a range of fynbos species. Plant Ecol..

[30] Liakoura V., Bornman J.F., Karabourniotis G. (2003). The ability of abaxial and adaxial epidermis of sun and shade leaves to attenuate UV-A and UV-B radiation in relation to the UV absorbing capacity of the whole leaf methanolic extract. Physiol. Plant..

[31] Guo R., Yuan G., Wang Q. (2011). Effect of sucrose and mannitol on the accumulation of health-promoting compounds and the activity of metabolic enzymes in broccoli sprouts. Sci. Hortic..

[32] Tsormpatsidis E., Henbest R.G.C., Davis F.J., Battey N.H., Hadley P., Wagstaffe A. (2008). UV irradiance as a major influence on growth, development and secondary products of commercial importance in Lollo Rosso lettuce ‘Revolution’ grown under polyethylene films. Env. Exp. Bot..

[33] Ruiz J.M., Rivero R.M., Lopez-Cantarero I., Romero L. (2003). Role of Ca2+ in metabolism of phenolic compounds in tabacco leaves (*Nicotiana tabacum* L.). Plant Growth Regul..

[34] Coley P.D., Bryant J.P., Chapin F.S. (1985). Resource availability and plant antiherbivore defense. Science.

[35] Ibrahim M.H., Jaafar H.Z.E. (2011). Enhancement of leaf gas exchange and primary metabolites, under carbon dioxide enrichment up-regulate the production of secondary metabolites of *Labisia pumila* seedlings. Molecules.

[36] Shure D.J., Wilson L.A. (1993). Patch-size effects of plant phenolics in successful openings of the Southern Appalachians. Ecology.

[37] Agnaniet H., Menut C., Bessie J.M. (2004). Aromatic plants of tropical central Africa. Part XLIX: Chemical composition of essential oils of the leaf and rhizomes of *Aframomum giganteum* from Gabon. Flavour Fragrance J..

[38] Katsube T., Tabata H., Ohta Y., Yamasaki Y., Anuurad E., Shiwaku K. (2004). Screening for antioxidant activity in edible plant products: Comparison of low-density lipoprotein oxidation assay, DPPH radical scavenging assay and Folin-Ciocalteu assay. J. Agric. Food Chem..

[39] Mosaleeyanon K., Zobayed S.M.A., Afreen F., Kozai T. (2005). Relationships between net photosynthetic rate and secondary metabolite contents in St. John’s wort. Plant Sci..

[40] Fonseca J.M., Rushing J.W., Rajapakse N.C., Thomas R.L., Riley M.B. (2007). Potential implications of medicinal plant production in controlled environments: The case of feverfew (*Tanacetum parthenium*). HortScience.

[41] Low P.S., Merida J.R. (1996). The oxidative burst in plant defense: Function and signal transduction. Physiol. Plant..

[42] Jabs T., Tschope M., Colling C., Hahlbrock K., Scheel D. (1997). Elicitor-stimulated ion fluxes and O2 from the oxidative burst are essential components in triggering defense gene activation and phytoalexin synthesis in parsley. Proc. Natl. Acad. Sci. USA.

[43] Yuan Y.J., Li C., Hu Z.D., Wu J.C. (2001). Signal transduction pathway for oxidative burst and taxol production in suspension cultures of *Taxus chinensis* var. mairei induced by oligosaccharide from *Fusarium oxysprum*. Enzyme Microb. Technol..

[44] Berglund T., Ohlsson A.B. (1995). Defensive and secondary metabolism in plant tissue cultures, with special reference to nicotinamide, glutathione and oxidative stress. Plant Cell Tissue Organ Cult..

[45] Gregoriou K., Pontikis K., Vemmos S. (2007). Effects of reduced irradiance on leaf morphology, photosynthetic capacity, and fruit yield in olive (*Olea europaea* L.). Photosynthetica.

[46] Wei S.L., Wang W.Q., Chen X.H., Qin S.Y., Chen X.T. (2005). Studies on the shade-endurance capacity of *Glycyrrhiza uralensis*. Zhongguo Zhong Yao Za Zhi.

[47] Fan Y., Wang Y., Tan R., Zhang Z. (1998). Seasonal and sexual variety of ginkgo flavonol glycosides in the leaves of *Ginkgo biloba* L. Zhongguo Zhong Yao Za Zhi.

[48] Ibrahim M.H., Jaafar H.Z.E., Rahmat A., Zaharah A.R. (2011). The relationship between phenolics and flavonoid production with total non structural carbohydrate and photosynthetic rate in *Labisia pumila *Benth. Under high CO_2_ and nitrogen fertilization. Molecules.

[49] Winger A., Purdy S., Maclean A., Pourtau N. (2006). The role of sugars in integrating environmental signals during the regulation of leaf senescence. New Phytol..

[50] Lambers H. (1993). Rising CO_2_, secondary plant metabolism, plant-herbivore interactions and litter decomposition. Plant Ecol..

[51] Reddy A.R., Reddy K.R., Padjung R., Hodges H.F. (1996). Nitrogen nutrition and photosynthesis in leaves of pima cotton. J. Plant Nutr..

[52] Cronin G., Lodge D.M. (2003). Effects of light and nutrient availability on the growth, allocation, carbon/nitrogen balance, phenolic chemistry, and resistance to herbivory of two freshwater macrophytes. Oecologia.

[53] Ibrahim M.H., Jaafar H.Z.E., Haniff M., Yusop R. (2010). Changes in the growth and photosynthetic patterns of oil palm (*Elaeis guineensis* Jacq.) seedlings exposed to short term CO_2_ enrichment in a closed top chamber. Acta Physiol. Plant.

[54] Afreen F., Zobayed S.M.A., Kozai T. (2005). Spectral quality and UV-B stress stimulate glycyrrhizin concentration of *Glycyrrhiza uralensis* in hydroponic and pot system. Plant Physiol. Biochem..

[55] Suza R., Valio I.F.M. (2003). Leaf optical properties as affected by shade in samplings of six tropical tree species differing in succesional status. Braz. J. Plant Physiol..

[56] Meyer S., Cerovic Z.G., Goulas Y., Montpied P., Demotes S., Bidel L.P.R., Moya I., Dreyer E. (2006). Relationship between assessed polyphenols and chlorophyll contents and leaf mass per area ratio in woody plants. Plant Cell Environ..

[57] Margna U., Margna E., Vainjarv T. (1989). Influence of nitrogen nutrition on the utilization of L-phenylalanine for building flavonoids in buckwheat seedling tissues. J. Plant Physiol..

[58] Ibrahim M.H., Hawa Z.E.J. (2011). Carbon dioxide fertilization enhanced antioxidant compounds in malaysian kacip fatimah (*Labisia pumila* Blume). Molecules.

[59] Ibrahim M.H., Jaafar H.Z.E. (2011). The influence of carbohydrate, protein and phenylanine ammonia lyase on up-regulation of production of secondary metabolites (Total phenolics and flavonoid) in *Labisia pumila* (Blume) fern-vill (Kacip Fatimah) under high CO_2_ and different nitrogen levels. Molecules.

[60] Thayumanam B., Sidasivam S. (1984). Carbohydrate chemistry. Qual. Plant Foods Hum. Nutr..

[61] Tognetti R., Johnson J.D. (1999). The effect of elevated atmospheric CO_2_ concentration and nutrient supply on gas exchange, carbohydrates and foliar phenolics concentration in live oak (*Quercus virginiana* Mill.) seedlings. Ann. For. Sci..

[62] Alexieva V., Sergiev S., Karanov E. (2001). The effect of drought and ultraviolet radiation on growth and stress markers in pea and wheat. Plant Cell Environ..

[63] Heath R., Packer L. (1968). Photoperoxidation in isolated chloroplasts. I. Kinetics and stoichiometry of fatty acid peroxidation. Arch. Biochem. Biophys..

[64] Ibrahim M.H., Jaafar H.Z.E., Haniff M.H., Raffi M.Y. (2010). Changes in growth and photosynthetic patterns of oil palm seedling exposed to short term CO_2_ enrichment in a closed top chamber. Acta Physiol. Plant..

[65] Ibrahim M.H., Jaafar H.Z.E. (2011). The relationship of nitrogen and C/N on secondary metabolites and antioxidant activities in three varieties of Malaysia kacip fatimah (*Labisia pumila* Blume). Molecules.

[66] Ibrahim M.H., Jaafar H.Z.E. (2011). Photosynthetic capacity, photochemical efficiency and chlorophyll content of three varieties of *Labisia pumila* benth. Exposed to open field and greenhouse growing conditions. Acta Physiol. Plant..

[67] Ibrahim M.H., Jaafar H.Z.E., Rahmat A., Zaharah A.R. (2011). Effects of nitrogen fertilization on synthesis of primary and secondary metabolites in three varieties of kacip fatimah (*Labisia pumila* Blume). Int. J. Mol. Sci..

[68] Idso S.B., Kimball B.A., Hendrix D.L. (1996). Effects of atmospheric CO_2_ enrichment on chlorophyll and nitrogen nutrition concentrations of four sour orange tree leaves. Environ. Exp. Bot..

